# Supplementary material for: A Humanized Yeast Phenomic Model of Deoxycytidine Kinase to Predict Genetic Buffering of Nucleoside Analog Cytotoxicity
Source: Genes (Basel). 2019 Sep 30;10(10):770. doi: 10.3390/genes10100770 (PMC6826991; doi:10.3390/genes10100770)
Supplement: Supplementary file 1 [file genes-10-00770-s001.zip › Additional_Files/Additional_File7_GOTermHeatmaps.zip!/Additional_File7_GOTermHeatmaps/cytosolic_small_ribosomal_subunit.pdf]

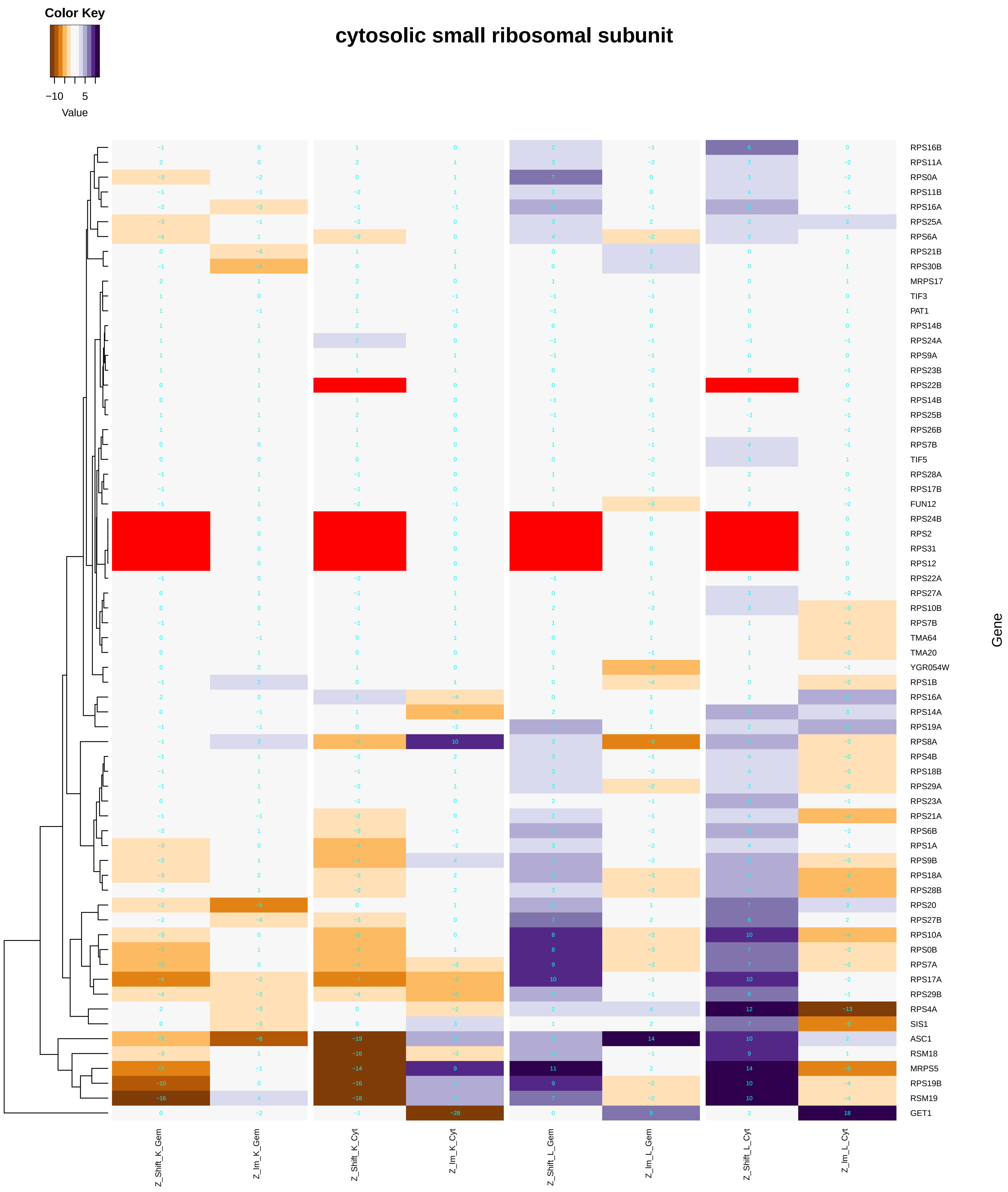

cytosolic small ribosomal subunit
Color Key
−10
5
Value
RPS16B
RPS11A
RPS0A
RPS11B
RPS16A
RPS25A
RPS6A
RPS21B
RPS30B
MRPS17
TIF3
PAT1
RPS14B
RPS24A
RPS9A
RPS23B
RPS22B
RPS14B
RPS25B
RPS26B
RPS7B
TIF5
RPS28A
RPS17B
FUN12
RPS24B
RPS2
RPS31
RPS12
RPS22A
RPS27A
RPS10B
RPS7B
TMA64
TMA20
YGR054W
RPS1B
RPS16A
RPS14A
RPS19A
RPS8A
RPS4B
RPS18B
RPS29A
RPS23A
RPS21A
RPS6B
RPS1A
RPS9B
RPS18A
RPS28B
RPS20
RPS27B
RPS10A
RPS0B
RPS7A
RPS17A
RPS29B
RPS4A
SIS1
ASC1
RSM18
MRPS5
RPS19B
RSM19
GET1
Gene
Z_Shift_K_Gem
Z_Im_K_Gem
Z_Shift_K_Cyt
Z_Im_K_Cyt
Z_Shift_L_Gem
Z_Im_L_Gem
Z_Shift_L_Cyt
Z_Im_L_Cyt
